# Atlanta Society of Medicine

**Published:** 1887-03

**Authors:** 


### Society Reports.

### ATLANTA SOCIETY OF MEDICINE.

Stated Meeting, January 18, 1887.

Dr. Virgil O. Hardon, President, in the chair.

The subject for discussion,

OPHTHALMIA NEONATORUM, was opened by Dr. A. W. Calhoun. Dr. Calhoun said: Ophthalmia neonatorum produces, perhaps, more blindness in children than all other diseases combined. It is estimated that forty-six per cent. of all cases of blindness in the asylums and academies for the blind in the country result from this cause. Three-fourths of all the cases in the Georgia Academy for the Blind are the result of this disease. The first symptoms of the disease usually appear on the third or fourth day. Among the first symptoms is profuse lachrymation, the discharge in a short time becoming muco-purulent and then rapidly purulent. Great swelling of the upper lids comes on in a short time after the inflammation is well set up, but the child usually seems to suffer but little. The great danger to be apprehended is sloughing of the cornea, which frequently occurs where proper and prompt attention is not given the eye. The absence of pain may be accounted for, when the cornea sloughs, by the destruction of the nerves of the cornea, which are destroyed by the sloughing process. Among the earliest symptoms of corneal complication is its haziness, which, if neglected, runs rapidly to a thick deposit.

In speaking of the causes of the disease, Dr. Calhoun said the chief cause was inoculation from the uterine or vaginal discharges which came in contact with the eye during the birth of the child. This discharge might or might not be a gonorrhœal discharge. He also said that exposure to bright lights sometimes caused the disease, and irritating soaps getting into the child's eyes while it was being bathed, he thought, might produce it. He said as a proof of the statement that inoculation was the cause of the disease, the per cent. of cases was always much less in those cases in which the vagina of the mother had been thoroughly cleansed of all discharges just prior to giving birth to the infant. He said if there was any difference between ophthalmia in the new-born, caused by gonorrhœal discharges and those cases brought on from other causes, it was that the cases produced by gonorrhœal virus were more violent. Clinically there was no difference.

### TREATMENT.

Dr. Calhoun said, in speaking of the treatment, that persistent cleanliness is the first great indication. The eye should be thoroughly cleansed every half hour while the discharge is profuse; but even later on under no circumstances should it go longer than two hours without being cleansed. He advised for this purpose astringent and antiseptic washes. He mentioned boracic acid, ℈j to ℥j, bichloride of mercury, half grain to six ounces of water; alum, three or four grains to the ounce, makes a valuable wash for cleansing the eye. He advised that during the cleansing process the child's head should be held between the knees. Dr. Calhoun said that he never used sponges in cleansing, but small pieces of old soft linen, always burning it as soon as used. Never use the same cloth the second time. He cautions every one as to the contagiousness of the disease, saying that it had been proved that the discharge from the eye of a child suffering with the disease, after being diluted 1 to 10,000, still retained more or less of its contagious properties.

When the cornea was to be examined he advised the use of retractors to prevent any pressure on the cornea, which may produce rupture and allow the escape of the lens, should there be a deep corneal ulcer. This accident had happened in his own practice in one case.

The sheet-anchor in the treatment of the disease was a solution of nitrate of silver, ten grains to the ounce, applied with a camel hair brush, and immediately neutralized by chloride sodium. In order to apply this properly the lids should be everted and the nitrate of silver solution applied to the conjunctiva by means of a brush. He advises that the lids be brushed over at once, after the nitrate of silver has been used, with a solution of chloride of sodium, which, in a measure, neutralizes the effects of the silver solution. In addition to the nitrate of silver he recommends the following to be used by the nurse in the absence of the medical attendant:

℞. Zinc sulph....................
Alum........................ aa grs. j.
Aq. camph.................... ℥i.
M. Sig. To be instilled into the eye three times daily.

Cloths wrung out of ice-cold water and applied frequently, but for a short time, to the eyes will prove beneficial in the acute stage.

If the cornea becomes affected, then apply a few drops of the following:

℞. Atropia sulph....................grs. ss.
Aq. Dest...................... ℥i.
M.

If by this means you succeed in dilating the pupil, you generally save the cornea.

The disease runs its acute course in ten days to two weeks if left alone.

Dr. Gaston said: The point that impresses me is the prophylactic treatment. The statistics of some of the European hospitals show that when a weak solution of nitrate of silver has been used in the eyes from birth, this disease has rarely occurred. If the gonorrhœal discharge, as a cause of the disease, is ruled out, what other discharges cause it?

Dr. Baird said the difficulty of making an examination, or even applying remedies, is well known. He suggested that when an examination was to be made, the child's arms and body be wrapped in a shawl; it could be more firmly held in this way. He said when in need of an elevator for raising the lids he used a hair-pin, bending the convexed end of the pin laterally, forming a very convenient elevator. The practice of large hospitals cannot be followed and their statistics relied on in private practice. The practice of douching the vagina with astringent washes before delivery for the prevention of the disease might do for hospitals where there was a peculiar susceptibility to the disease. The profuse lubricating discharge is very helpful in labor, and he opposed the use of astringents before labor as a prophylactic.

Dr. Crawford asked how many of these cases get well.

Dr. Calhoun, in closing the discussion, said he could not answer the question of how many get well, as many are never treated. All cases if properly treated in the beginning ought to get well. He does not think that all women have purulent discharges, for if they did ninety-nine out of a hundred children would have the disease under discussion. In syringing out the vagina great harm can be done. There is no special need of using astringents and antiseptics ; a simple wash will do unless the former is indicated for some special reason. Use the solution of nitrate of silver in the eye once a day. Can use it weaker, but thinks the 2 per cent. solution the best. Never uses the sugar of lead, because when the cornea is ulcerated it leaves lead deposits which will always remain. He does not think that counter-irritation on the temples does any good.

